# The academic impact of Open Science: a scoping review

**DOI:** 10.1098/rsos.241248

**Published:** 2025-03-05

**Authors:** Thomas Klebel, Vincent Traag, Ioanna Grypari, Lennart Stoy, Tony Ross-Hellauer

**Affiliations:** ^1^ Open and Reproducible Research Group, Know Center Research GmbH, Graz, Austria; ^2^ Centre for Science and Technology Studies (CWTS), Leiden University, Leiden, The Netherlands; ^3^ Athena Research Center, Marousi, Greece; ^4^ OpenAIRE, Athens, Greece; ^5^ Technopolis Group, Brussels, Belgium

**Keywords:** academic impact, Open Science, Open Access, FAIR Data, Citizen Science, scoping review

## Abstract

Open Science seeks to make research processes and outputs more accessible, transparent and inclusive, ensuring that scientific findings can be freely shared, scrutinized and built upon by researchers and others. To date, there has been no systematic synthesis of the extent to which Open Science (OS) reaches these aims. We use the PRISMA scoping review methodology to partially address this gap, scoping evidence on the academic (but not societal or economic) impacts of OS. We identify 485 studies related to all aspects of OS, including Open Access (OA), Open/FAIR Data (OFD), Open Code/Software, Open Evaluation and Citizen Science (CS). Analysing and synthesizing findings, we show that the majority of studies investigated effects of OA, CS and OFD. Key areas of impact studied are citations, quality, efficiency, equity, reuse, ethics and reproducibility, with most studies reporting positive or at least mixed impacts. However, we also identified significant unintended negative impacts, especially those regarding equity, diversity and inclusion. Overall, the main barrier to academic impact of OS is lack of skills, resources and infrastructure to effectively re-use and build on existing research. Building on this synthesis, we identify gaps within this literature and draw implications for future research and policy.

## 1. Introduction

In this paper, we aim to scope and summarize existing evidence on the academic impact of Open Science. Open Science seeks to make research processes and outputs more accessible, transparent and inclusive, ensuring that scientific findings can be freely shared, scrutinized and built-upon by researchers and others [1]. Key practices include Open Access publishing (OA), where articles are made freely available to readers [2–4], open (or FAIR, meaning findable, accessible, interoperable and re-usable) sharing of data and code [5–8], transparency of processes and methods [9–12], transparent models of peer review [13,14] and engaging publics and others beyond academia (e.g. civil society, policymakers, industry) in participatory research processes [15–17].

In promoting these distinct practices, the Open Science (OS) movement has developed as a synthesis of what Field [18] rightfully terms ‘a constellation of communities, rather than a single, homogeneous one’ (p. 149). Open Science’s roots can be found in the foundational Open Access statements of Budapest [4], Berlin [19] and Bethesda [20], all focusing on access to knowledge and highlighting OA’s potential to democratize and increase use of knowledge. Rising awareness of poor levels of robustness of results [21], labelled a ‘reproducibility crisis’ in some disciplines in the mid-2010s [22], also led to emphasis on transparency of methods and reporting, as well as availability of research materials. The latter has been further boosted by a focus on enabling reuse of research data, code and other materials, most notably via the FAIR agenda among researchers and infrastructure providers [8,23]. Meanwhile, quasi-independent movements towards Citizen Science (CS) and participatory research have tended to be incorporated under the umbrella of OS, given alignment of aims such as public engagement and participation [24,25].

This thumbnail sketch already indicates that the aims of Open Science are far from uniform. Reviewing the OS literature in 2014, Fecher & Friesike [26] identified five ‘schools of thought’ in advocating for Open Science: to make knowledge freely available, increase efficiency of knowledge production, create open infrastructures and tools, make science accessible for the public, and develop alternative systems for evaluation and assessment. Since then, the picture has only become more complex (for example, a notable omission in the Fecher & Friesike paper is transparency, quality and robustness of methods and results). Recognizing such difficulties, UNESCO [24] attempted to establish global consensus on the OS programme, detailing principles including transparency, equality, responsibility, collaboration, flexibility and sustainability, and citing quality and integrity, collective benefit, equity and fairness, and diversity and inclusiveness as core values.

As a constellation of communities with often distinct motivations, and requiring support and action from various actors with each their own priorities (research communities, research performing organizations, research funding organizations, publishers, governments, as well as individual researchers), OS has what we recognize as an ‘interpretative flexibility’ [27]. Its complex meanings and interpretations among different groups need not always align, and along the various routes to implementation, unintended consequences may be expected [28,29]. This complexity, along with the heavy investments required to realize OS, necessitate careful monitoring. Moreover, OS as a transformative change may require not only monitoring of new indicators, but a different monitoring approach altogether [30]. Yet thus far, large-scale monitoring of OS (e.g. the EC’s Open Science Monitor [31], and various national monitors [32–34]) seem mainly focused on understanding the spread of open practices (uptake), rather than whether that uptake is having the downstream real-world effects envisioned by the various manifestos and declarations made by OS communities. Understanding the latter is essential for policymakers, stakeholders and researchers to evaluate the effectiveness of activities and to make informed decisions.

Understanding the real-world change resulting from projects or programmes is the task of impact evaluation. We define impact as ‘long-lasting, elementary and wide-spread change’ upon academia, society or the economy which may be either ‘direct or indirect, intended or unintended, [and] relate to behavioural and/or systemic changes’ [35]. In the realm of policy evaluation, impact usually denotes population level effects, which result from outputs and intermediary outcomes of a given intervention [36]. Mapping the chain of effects from intervention to impacts is done through frameworks such as intervention logics, which aim to explicate the causal mechanisms at play.

In this scoping review, we consolidate evidence to date for the academic impacts of Open Science. We define academic impact with Ravenscroft *et al*. as ‘the impact that scientific research has within the academic sphere’ [37], hence distinguishing it from broader impacts upon society or the economy.^1^ Related to the above discussion, we consider the effects of Open Science on research quality (standards of research processes and products), trust and integrity (i.e. honest and verifiable methods), equity (fairness, equal participation, minimizing biases), collaboration (work among research groups), efficiency and productivity (increased speed or economic effectiveness), reproducibility, reuse of research outputs, as well as citations (included since citations are often taken as direct indicators of academic impact).

We follow the PRISMA Extension for Scoping Reviews methodology (PRISMA-ScR) [39] to scope, appraise, summarize and synthesize knowledge from existing literature demonstrating the academic impact of OS in general, as well as that of its constituent aspects: OA, Open and FAIR Data (OFD), Open Methods, Open Code/Software, Open Evaluation and CS. Given this is a scoping (not systematic) review, we do not aim to provide a systematic assessment of certain effects and weigh the evidence and critically assess questions around causal inference. Nonetheless, we appraise the literature and provide indications of strengths and weaknesses, also in terms of causality.

Our main research question (RQ1) is: what evidence exists in the literature regarding the effect of OS on the academic impact of research? Secondary research questions are:

— SRQ1: what types of positive or negative, direct or indirect academic impact are observed?
— SRQ2: what kinds of mechanisms produce them?
— SRQ3: what specific enabling and/or inhibiting factors (drivers and barriers) are associated with these impacts?
— SRQ4: what knowledge gaps emerge from this analysis?

## 2. Methods

To address these research questions, the study used the PRISMA framework to align study selection with the research question and followed the relevant aspects of the PRISMA Extension for Scoping Reviews (PRISMA-ScR) to ensure thorough mapping, reporting and analysis of the literature [39]. The study proceeded in four phases: identifying potentially pertinent literature, screening and selecting for eligibility, data-charting included studies, and analysing and reporting our results. Our methodological protocol was pre-registered on 31 October 2022 [40], with an addendum further specifying processes for ‘snowball’ searching and identification of relevant ‘grey literature’ sources published on 29 June 2023 [41]. Changes to pre-registration are detailed in electronic supplementary material, S1.

### 2.1. Identification of relevant studies

Search was conducted for relevant peer-reviewed literature published in English from 1 January 2000 using Scopus and Web of Science.^2^ Relevant keywords were assembled and prioritized through search piloting prior to pre-registration (see electronic supplementary material, S1 for more details). The search strategy was developed iteratively. First, relevant high-level terms were defined for ‘impact’ generally (i.e. impact, effect, outcome) and academic impact specifically (i.e. efficiency, productivity, quality, education, reproducibility, reuse, citations, collaboration, equity, diversity and inclusion). From this, the following primary search strings for Web of Science (WoS) and Scopus database searches were specified (see table 1), with database queries run and search result retrieved on 2 November 2022. Across both databases a total 11 420 initial results were retrieved.

**Table 1.** Primary search strings for Web of Science (WoS) and Scopus. Explanation of abbreviations: TI = title, AB = abstract, TS = topic, TITLE-ABS = title and abstract, TITLE-ABS-KEY = title, abstracts and keywords.

Web of Science	[to be run in all databases, for time 01-01-2000 to 31-12-2022] (TI= (‘open scien*’ OR ‘science 2.0’ OR ‘open data’ OR ‘FAIR data’ OR ‘open access’ OR (‘open code’ OR ‘open software’ OR ‘open tool*’) OR ‘open method*’ OR ‘citizen science’ OR ‘open peer review’ OR ‘open metric*’) OR AB= (‘open scien*’ OR ‘science 2.0’ OR ‘open data’ OR ‘FAIR data’ OR (‘open code’ OR ‘open software’ OR ‘open tool*’) OR ‘open method*’ OR ‘citizen science’ OR ‘open peer review’ OR ‘open metric*’ OR ‘open access publ*’ OR ‘open access paper*’ OR ‘open access journal*’ OR ‘open access book*’)) AND TS = ((impact* OR effect* OR outcome*) AND (quality OR citation* OR integrity OR equi* OR collaborat* OR trust OR efficien* OR re-us* OR reus* OR productiv*))
Scopus	TITLE-ABS (‘open scien*’ OR ‘science 2.0’ OR ‘open data’ OR ‘FAIR data’ OR (‘open access’ W/1 publ* OR paper* OR journal* OR book*) OR (‘open code’ OR ‘open software’ OR ‘open tool*’) OR ‘open method*’ OR ‘citizen science’ OR ‘open peer review’ OR ‘open metric*’) OR TITLE (‘open access’) AND TITLE-ABS-KEY ((impact* OR effect* OR outcome*) AND (quality OR citation* OR integrity OR equi* OR collaborat* OR trust OR efficien* OR re-us* OR reus* OR productiv*)) AND (PUBYEAR> 1999) AND (LIMIT-TO (LANGUAGE , ‘English’))

The next phase involved identifying further literature via (i) ‘snowball’ search, i.e. analysing relevant citations to and from already included studies (via OpenAlex), and (ii) ‘grey literature’ search for non-peer-reviewed material from websites (including the EC, UNESCO, OECD, for example), preprint servers (OSF preprint search) and web search (Google). Full documentation, code and data for this phase are shared with the full dataset [42].

### 2.2. Selection of eligible studies

We used the PRISMA-ScR checklist (electronic supplementary material, S2) for the selection process. The following inclusion criteria were used for both title/abstract and full-text screening:

— Studies including evidence of the academic impact of OS^3^ (i.e. OA, Open/FAIR Data^4^, Open Methods, Open Code/Software, CS^5^, or Open Evaluation)
— Conducted internationally or nationally
— Published from 1 January 2000 until the date of search
— Text in English
— Full-text available
— Study is either a research article, review article, conference paper, or other peer-reviewed output, or a grey literature study from a recognized stakeholder
— All methodologies (quantitative, qualitative, mixed, etc.) eligible

The primary focus was on original studies. However, we included review articles where the synthesized findings enabled broader conclusions in the literature. For example, the inclusion of reviews on the Open Access Citation Advantage, where the literature is particularly dense.

For title/abstract screening, following an initial screening of the WoS/Scopus results to exclude clear false positives, results were merged and de-duplicated, leaving 4556 initial records. Titles and abstracts were then screened by two researchers, the first coding items either ‘yes’, ‘no’ or ‘unsure’ for inclusion, and the second reviewing all results judged ‘unsure’ to either include or exclude. The aspect of OS addressed by the study (whether Open Access, Open/FAIR data, etc.) was also recorded at this stage.

The full-texts of the 541 remaining studies were then sought using all available means (i.e. search via library log-in credentials, inter-library loans and emailing authors). A total of 541 full-texts were retrieved and stored in a shared folder using the open source Zotero reference management software. Full-texts were then screened and assessed for inclusion by one researcher, following which 301 total studies remained.

**Figure 1.**
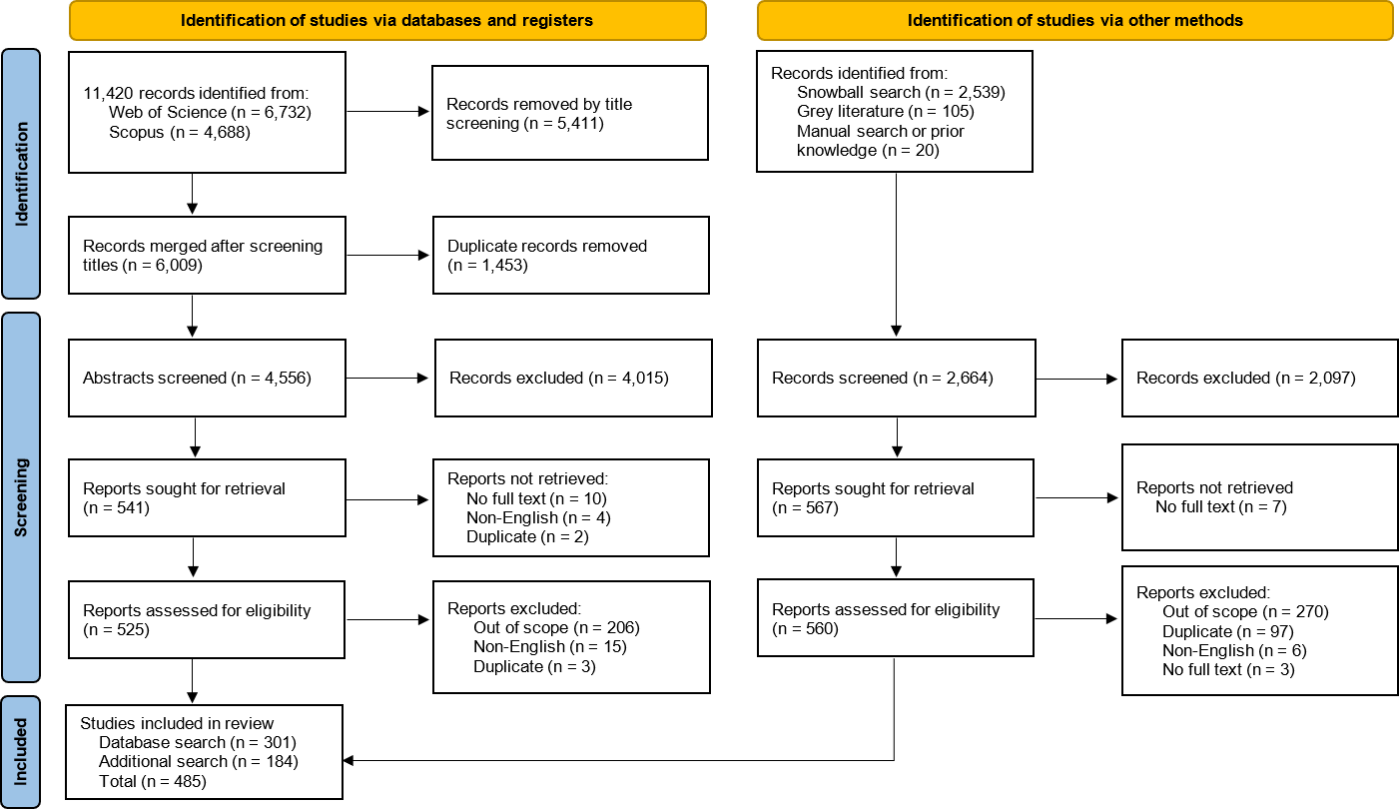
PRISMA-P chart of selection process.

Title/abstract screening of snowball and grey literature identified 2664 studies, reduced to 184 following full-text screening. Across all searches, 485 relevant studies were identified for inclusion in this Scoping Review. The full process of selection is summarized using the PRISMA-P chart below (figure 1).

### 2.3. Extraction of data

Studies were assigned to individual co-authors based on their relevance to specific aspects of OS. We used a collaborative spreadsheet, hosted via MS Teams, to extract data for specific items including data sources, study aims, types of impact studied, key findings, coverage and our confidence assessment. The data-charting categories used are below in table 2.

**Table 2.** Categories extracted from included studies in the data charting process.

data chart heading	description
author	name of author/s
date	date article sourced
title of study	title of the article or study
publication year	year that the article was published
publication type	journal, website, conference, etc.
DOI/URL	unique identifier
exclusion	out of scope, non-English, duplicate
justification	if a study was deemed to be out of scope, a justification had to be provided.
study details and design (if applicable)	type of study, empirical or review, etc.; notes on methods used in study (whether qualitative or quantitative, which population demographics studied, etc.)
types of data sources included	detail the data sources
study aims	overview of the main objectives of the study
relevance to which aspect of Open Science	Open Access, Open/FAIR Data, Open Methods, Citizen Science, Open Evaluation, Open Science General
relevance to which aspect of impact	quality, citations, integrity, equity, collaboration, trust, efficiency, productivity, reuse
key findings	noteworthy results of the study that contribute to the scoping review question(s)
coverage	optional field to note any relevant information about the level of coverage of the study, e.g. only specific countries, disciplines, demographics covered
confidence assessment	optional field to note any concerns about reliability/generalizability of findings (e.g. conflict of interest, potential biases, small sample sizes or other methodological issues) within the study

Periodic checks on quality and consistency of extraction were carried out by the lead author, with inconsistencies discussed in team meetings and revisions conducted based on this feedback. Part of this process involved the elaboration and re-categorization of our initial list of impacts (cf. table 2, ‘relevance to which aspect of impact’). We merged the terms ethics and integrity, as well as efficiency and productivity, since they refer to the same underlying concepts. Based on the extracted content, we also added the new category novelty, as well as expanding the scope of equity to equity, diversity, and inclusion.. See table 3 for the final list of categories and their definitions.

**Table 3.** Categories/definitions of academic impact used for data charting and synthesis.

category	definition
citations	changes (positive or negative) to the citation measures of research outputs (raw or standardized citations, citedness)
collaboration	changes (positive or negative) in cooperation or teamwork regarding the disciplinary, institutional, demographic or geographic composition of researchers in research activities
efficiency and productivity	changes (positive or negative) in terms of resources, time or effort required for research activities
equity, diversity and inclusion (EDI)	changes (positive or negative) to inclusion, fairness, diversity and justice in research, including: distribution of resources, opportunities or credit; addressing systemic inequalities/discrimination, and inclusion of diverse perspectives or populations
ethics and integrity	changes (positive or negative) in accountability, objectivity, fairness, responsibility, or adherence to ethical and professional standards/principles in research processes
novelty	changes (positive or negative) in the development of new ideas, approaches, discoveries or insights
quality	changes (positive or negative) to the rigour, relevance, reliability or soundness of scholarly processes and outputs
reproducibility	changes (positive or negative) in the reproducibility, replicability, robustness or generalizability of research findings
reuse	changes (positive or negative) in the further uptake and exploitation of research outputs (e.g. findings, data, code, methods, materials) for new research activities
trust	changes (positive or negative) within the academic community to confidence in the integrity, reliability or credibility of researchers, research processes or research findings

### 2.4. Collating, summarizing and reporting the results

The data on all included studies from the data-charting process is available at [42]. Following the data-charting process, the study team discussed main emergent themes including impact types and mechanisms. We then delegated responsibilities for drafting individual sections into our results below, summarizing the extracted information. These results are described in relation to the research question and in the context of the overall study purpose. Following the presentations of results, we present a final synthesis across all studies including gap identification of areas where further research is required. Finally, we conclude with a more general discussion of our findings and implications for future research and policy.

## 3. Results

**Figure 2.**
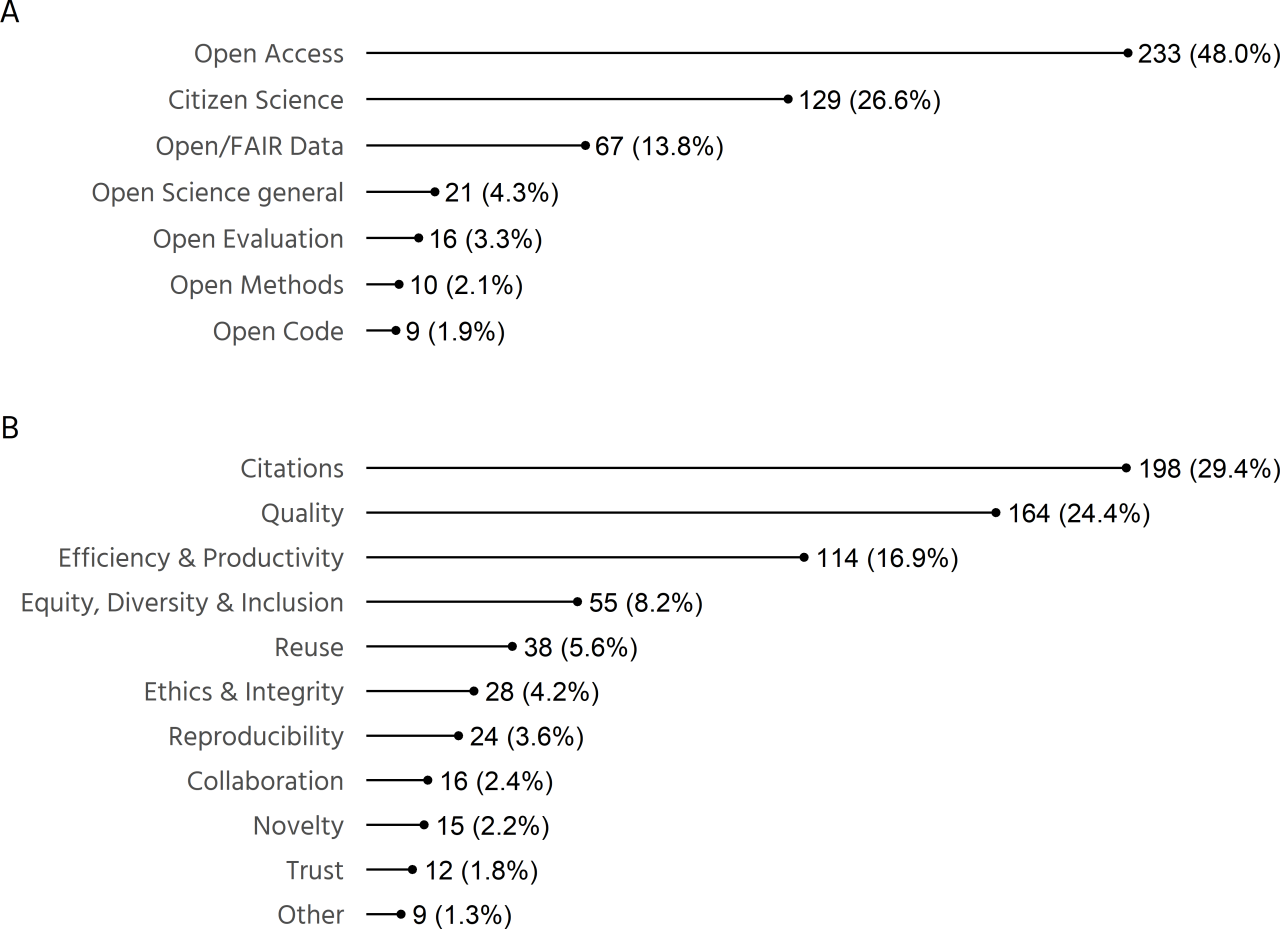
(A) Number of studies reporting academic impact of Open Science by relevance to aspects of Open Science. (B) Number of studies reporting academic impact of Open Science by type of impact. Note that the numbers reported in (B) do not sum to 485 because some studies reported multiple types of impact.

In this study, we systematically scoped the evidence to date regarding the academic impact of OS. Our analysis is based on 485 studies that provide evidence towards impacts of OS on academia. The largest share of the reviewed literature concerns the impact of Open Access (233 studies), followed by Citizen Science (129), Open/FAIR data (67). Fewer studies reported on the impacts of general forms of OS (21), Open Evaluation (16), Open Methods (10) and Open Code (9) (see figure 2A). The main type of impact reported concerns citations, but other types of impact, such as towards quality, efficiency and equity, were also found (figure 2B). Note that the sum of impact types is larger than the number of included studies, since some studies demonstrated multiple types of impact.

**Figure 3.**
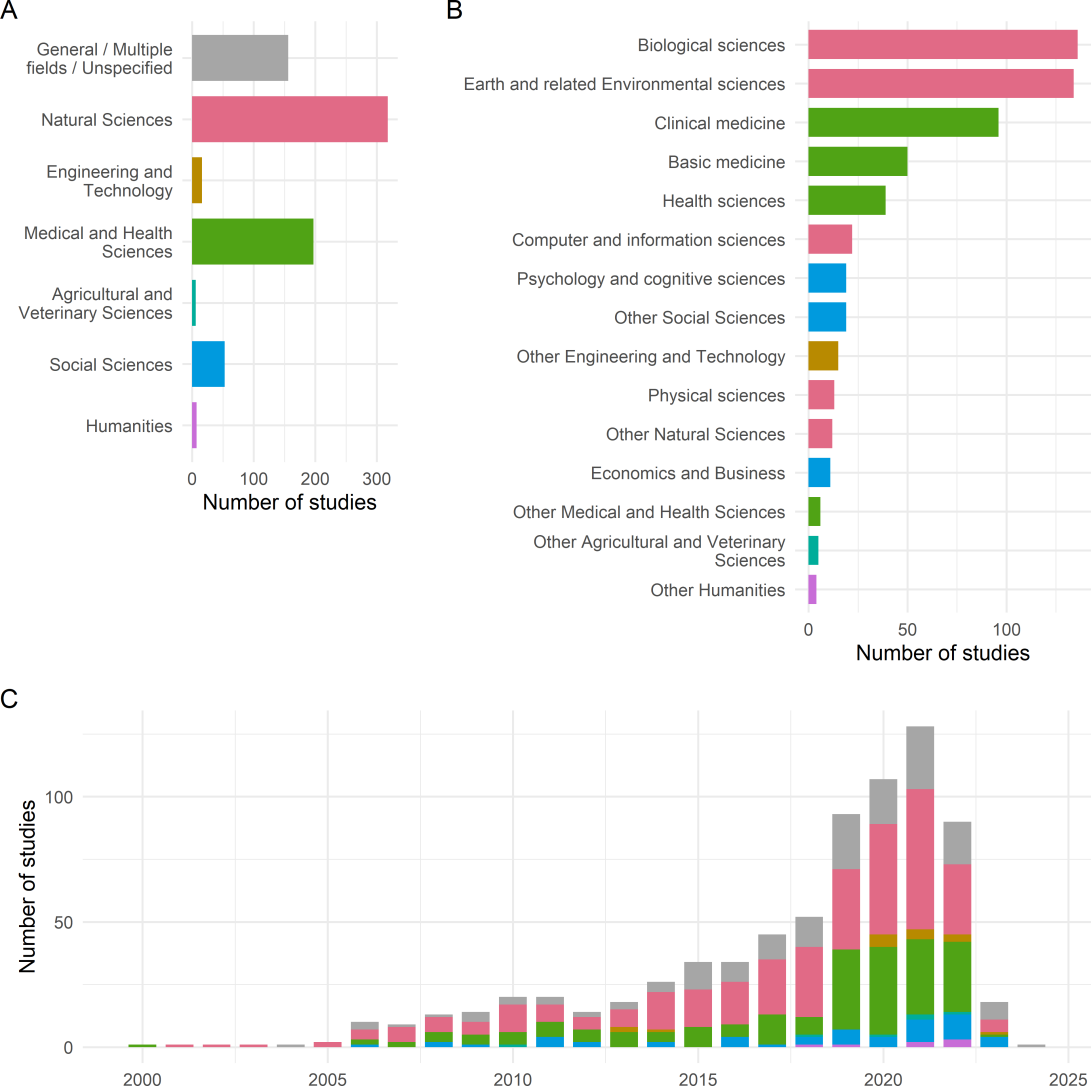
Disciplinary coverage and publication year of included studies. (A) Top-level Frascati classification. (B) Second-level Frascati classification. We collapsed categories with fewer than 10 included publications. (C) Publication year.

To provide further context, we also analysed the included studies according to their publication year, article type and the topic they covered (figure 3). The majority of studies investigated the impact of Open Science among research in the natural sciences, followed by the medical and health sciences, as well as cases covering multiple or unspecified fields. The median publication year was 2019, indicating a substantial growth of the literature in recent years, despite our main search only partially covering 2022. In terms of article types, the included studies were mostly research articles (84% of included articles), followed by review articles (8%), conference papers (4%) and other types of articles (preprints, book chapters and grey literature).

### 3.1. Open Access

Our review identified 233 relevant articles related to OA [44–276], making it the most studied element of OS for academic impact. Of these, 160 studies investigate effects on citations and the so-called Open Access Citation Advantage (OACA). Other areas of inquiry include the effect of OA on quality (40 relevant studies), equity, diversity and inclusion (37), efficiency and productivity (31) and other areas (20). In the following, we review the literature along the following dimensions: Open Access Citation Advantage, equity in OA publishing, preprints and other changes to the landscape of scholarly communication brought about by OA publishing.

#### 3.1.1. Open Access Citation Advantage

The effect of OA publishing on citations, especially regarding differences with closed access, has been studied extensively. A major discussion has been whether there exists an ‘Open Access Citation Advantage’ (henceforth OACA), the assumption being that easier access to literature leads to higher readership and subsequently higher citation counts. Given the large number of studies (160 studies identified as relevant to citations), we here mainly report on previously conducted reviews [103,150,209], complemented by more recent studies.

There is substantial heterogeneity in methodological approaches among the studies identified as relevant to the OACA in our review. Asai [209] identifies three types of approaches: (i) comparing measures of citations ‘between randomly sampled subscription and open access articles or journals’, (ii) employing econometric models with control variables, and (iii) examining ‘the changes in citation scores for individual journals that shifted from subscription to open access using time trend data’ (p. 108). In addition, most studies tend to focus on specific research fields or on certain modes of OA, typically using classifications like the following (cf. Piwowar *et al*. [266])^6^ :

— *Gold OA:* immediate free access with publication costs typically covered by authors or institutions
— *Green OA:* self-archiving of a free version in repositories, often with an embargo
— *Hybrid OA:* subscription-based journals offering optional paid open access for individual articles
— *Bronze OA:* free access provided by publishers without an explicit open license
— *Diamond OA:* immediate free access with no charges to authors or readers, funded by institutions or consortia

Beyond differences in general methodological approaches, there are also substantial differences in how the two key concepts, citations and Open Access status, are measured. Citation impact is measured in a variety of ways, comparing raw or standardized citations, or comparing journal impact factors between OA and non-OA journals, or comparing a number of further available indicators that rely on citations [209]. Similarly, approaches to determine whether a given article is OA differ substantially among the studies included in our review. Even in established databases such as Web of Science and Scopus, definitions and operationalizations of OA were unclear as of 2015 [176]. While the introduction of the Unpaywall service^2^ in 2017 (an open database of OA research articles), the highly cited article by Piwowar *et al*. [266] and the subsequent availability of Unpaywall’s data have led to some standardization, earlier studies relied on very different ways of defining and selecting individual publications or journals as being OA.

A key OACA study is the systematic review by Langham-Putrow *et al*. [150], which synthesizes evidence for and against the OACA. The authors report finding substantial heterogeneity in how studies aim to measure the OACA, for which reason they did not conduct a quantitative meta-analysis. Moreover, they found remarkably high levels of risk of bias. In their assessment, only three of 134 studies [88,224,277] were found to have a low risk of bias across the domains ‘population’, ‘data collection’, ‘study design’ and ‘results’. One of the three studies found to have a low risk of bias detected an OACA in general, one reported an OACA in subsets of data and one found no OACA. Disregarding risk of bias to report across all 134 relevant studies, the authors did identify an OACA in general, reporting that 47.8% of reviewed studies found an OACA, with the remaining studies finding no OACA (27.6%) or only in subsets (23.9%) with respect to journal, discipline or time. The authors did not find differences in how often studies reported an OACA by type of OA, but did find that studies with broader disciplinary coverage tended to find an OACA more often.

The degree of risk of bias across the literature surveyed by Langham-Putrow *et al*. [150] shows that causal effects are difficult to substantiate. The main sources for risk of bias reported by the authors were poorly described samples or samples insufficient to support conclusions, and missing justification for the choice of study period (e.g. length of citation window). In addition, a large number of confounding factors have been suggested to affect estimates of the OACA, which were also apparent in our review (e.g. ‘journal impact factor, number of authors, length of article, type of study’). Besides the confounding factors reported by Langham-Putrow *et al*., the studies identified in our review indicate further factors which might bias causal estimates of the OACA [103]:

— *Selection bias*: in the case of hybrid^7^ OA publishing, some claim that researchers might only choose to make their best works OA, which in turn would lead to increased citations based on publication quality, rather than OA status [130,236]. However, Gargouri *et al*. [216] find the OACA to be independent of self-selection, thus questioning the existence of this bias.
— *Early-view bias*: another hypothesized effect is the early-view effect on citations. Publications made available as preprints (i.e. prior to publication in a peer-reviewed journal) might receive more citations, since citations usually take some time to accrue [236].
— *Research funding*: research emanating from publicly funded projects might be of higher quality than the average research conducted in a field, due to selection effects at proposal stage. Since research funders often mandate OA, not controlling for funding could introduce spurious effects [113].
— *Journal prestige*: a common approach in studies assessing the OACA is to compare journal impact factors between OA and non-OA journals. However, these studies often do not control for other factors influencing impact factors, such as the extent to which journals are established and central to subdisciplines. Comparing new (OA) with established (non-OA) journals regarding their citation performance ignores these aspects.
— Other confounding factors discussed in the literature, such as the ‘absence of an appropriate control group of non-OA articles’ are summarized by Rovira-Esteva *et al*. [168].

Given this complexity, a clear evidence gap seems to be the lack of any systematic account of the causal pathways related to the OACA. A systematized model of known and potential causal pathways might bring clarity to the study of the OACA and inform design choices for future studies.

A shortcoming of the review by Langham-Putrow *et al*. [150] is the question of differences in OACA between modes of OA (hybrid, green, gold, bronze). Comparing outcomes on whether the OACA exists or not across OA modalities, Langham-Putrow *et al*. report finding no statistically significant effect. In their analysis, Langham-Putrow *et al*. merged hybrid and gold OA, because they report the two modes to be ‘sometimes conflated within the included studies’. Our review of the literature suggests that merging gold and hybrid OA masks an important difference, where hybrid articles tend to receive more citations than their closed counterparts, whereas gold articles tend to receive fewer.

To substantiate these claims, we conducted a post hoc analysis of all studies we identified which relate to the OACA. Given the complexities in assigning studies to different modes of OA and the lack of a thorough protocol, we provide a broad summary of our findings but no exact counts of outcomes.

The majority of studies find that *gold OA* journals have lower impact factors than closed-access journals (see for example [198,199,204,266]). This is especially salient in studies which compare multiple OA modes, where many find an OACA for hybrid and/or green OA, but not gold OA. However, the low citation rates of gold OA might be partly driven by other factors than OA, most prominently the fact that many gold OA journals are relatively new and thus have had less time to build reputation than closed-access journals [176].

By contrast, our analysis indicates that the majority of studies comparing citations received between *hybrid OA* and closed articles from the same journals find an OACA (see for example [85,137,262]; but see [140]). However, this effect might be partially driven by selection bias and effects of research funding.

Finally, studies assessing citations towards *green OA* articles tend to report finding an OACA (see for example [133,170,199,257,266,271]).

Taken together, we find clear evidence for differences in the OACA between modes of OA. Our results suggest that it might be futile to investigate and claim an overall OACA, but that efforts should probably focus on context-specific accounts of an OACA.

Lastly, the studies included in our review also reveal vastly different estimates of the size of the OACA. Studies with more elaborate methods that aim to make estimates of causal effects, such as the one by Staudt [156], tend to report low estimates of the OACA (in this case: 3%), compared with much larger estimates in other studies [85,170,225].

#### 3.1.2. Equity in Open Access publishing

Prominent among financial models for OA publishing are author-facing charges, so-called article processing charges (APCs). This business model has been identified as being a threat to equity in publishing very early [259], but is still widespread today [70,160]. APCs have been found to be positively correlated with journal metrics [54,59,60,62,75,131,218], although the effect is not present in all fields [178]. This inequity is further aggravated by APCs due to global differences in purchasing power parity [46], restricting publishing in high-impact journals to those with sufficient funding. Waivers have been found to be ineffective at countering this issue, in particular for researchers from lower- and upper-middle income countries [68,158].

Investigating the difference in geographic diversity of authors between closed and OA journals, Smith *et al*. [65] report lower geographic diversity among authors in OA journals, suggesting a prohibitive effect of publication costs for scientists from the Global South. By contrast, there is evidence that OA is improving equity in readership. Analysing a large bibliographic dataset from 2010 to 2019, Huang *et al*. [182] report finding higher diversity of citations in terms of (among others) institutions, countries and fields of research for OA publications.

Overall, the dynamics around APC-based publishing create a barrier for researchers aiming to publish their work, stratifying global publishing even further and undermining initial goals of the OA movement in terms of democratization [223]. Knöchelmann [234] discusses these trends against the objective of Open Access to democratize access to knowledge, arguing that dominant solutions in the form of Global North-driven OA policies create an accessibility problem and therefore publication injustices for authors from other regions. Thus, even though OA increases equity in terms of physical access, it has been found to decrease equity on the side of publishing research.

#### 3.1.3. Preprints

Another innovation aligned with Open Science is the use of preprints (public posting of non-peer-reviewed manuscripts, usually in advance of journal submission). Although preprinting has been common in disciplines like Physics (via the arXiv preprint server, established in 1991), rates have expanded rapidly across disciplines in recent years, especially during the COVID-19 pandemic [93,269,270]. We identified 14 studies reporting impacts on efficiency, quality and citations.

The use of preprints aims to enhance the *efficiency* (i.e. speed) of scholarly communication. Rates of improvement in dissemination time differ across disciplines, however. Abdill & Blekhman [270] reported that around two-thirds of *bioRxiv* preprints were eventually published in peer-reviewed journals, with a median time of around five and a half months. Studying *arXiv* preprints, Larivière *et al*. [63] reported a similar lag in Physics, but significantly longer times in Mathematics of over a year. Wang *et al*. [197] confirmed the latter, finding an average lag of 16.3 months in three subdisciplines of Mathematics between preprinting on arXiv and eventual article publication. The emergency response to the COVID-19 pandemic significantly reduced time lag, however, as Oikonomidi *et al*. [74] report a median lag of just two months for bioRxiv preprints on COVID-19 interventions.

Seven studies assess the *quality* of preprints compared with their published versions. Most focus on biomedical research, especially that related to the COVID-19 pandemic. Overall, these studies demonstrate that preprints which are eventually published within journals change relatively little, and are of similar or only somewhat lower quality across a range of outcome measures. These include: basic differences in text [92]; epidemiological estimates and expert assessment of quality in COVID-19 research [210]; study characteristics, outcomes and interpretative ‘spin’ [99]; validity of COVID-19 parameter estimations [228]; quality of reporting [93]; and numbers of figures/tables and conclusions reported [269]. Oikonomidi *et al*. [74] found, however, that one in six COVID-19 preprints did have at least one significant change in evidence reporting in their published version. In interpreting this evidence, it should be kept in mind that the included studies do not include comparison with preprints which did not later go on to be published (for obvious reason). This potentially leads to an overestimation of preprint quality (selection bias), where non-published preprints would presumably tend to be of lower quality on average.

Lastly, across various fields, published articles that were preprinted are associated with increased levels of citations compared with articles where no preprint was made available [205,230,250,264]. This literature makes no claims for direct causality, however, with potential for confounding factors like that those who preprint may be more likely to publish OA, or be generally more active in self-promotion, or that works of perceived higher potential impact are more likely to be preprinted.

#### 3.1.4. Other changes in the scholarly publishing landscape

The advent of OA publishing has been accompanied by multiple changes in the landscape of scholarly communication and publishing (see e.g. Eysenbach [80]). While the internet and modes of online publication have enabled OA, the move to OA publishing has in turn led to new forms of publishing, such as OA mega-journals, predatory publishing and an uptake in preprinting, which in turn has been sparking new modes of organizing peer review (see e.g. F1000, or eLife from 2023).

A total of 33 studies assessed impacts on quality in scientific publishing. Due to lack of basic editorial practices, predatory publishing is by definition expected to lead to publications of lower quality. While empirical investigations of articles published in potentially predatory journals indeed find them to be of lower quality [175,194], these articles have been found to accrue much fewer citations than comparable articles, and therefore have a low impact on science [138]. The use of blacklists (such as Beall’s list) to identify predatory journals has been subject of substantial debate and can be considered a secondary impact of OA publishing on equity, given its purported effect on ‘divisiveness, discrimination and stigmatization’ [278].

Beyond predatory publishing, multiple studies have analysed the impact of OA publishing on article quality. While acceptance rates have been found to be higher among OA journals [152], there is conflicting evidence as to differences in actual article quality. Some authors have found no difference in quality between OA and non-OA articles [58,135,202], while others report higher quality among non-OA articles [96]. In addition, evidence on the effect of OA mega-journals on article quality is sparse [190]. Retraction rates have been found to be higher among OA mega-journals [151], but this is arguably related to better editorial practices rather than lower quality research, as indicated by the finding that OA journals provide more detailed information on the reason for retractions [78].

In addition, the move to OA publishing has changed the business models of publishers. Quantifying costs and benefits of OA business models and self-archiving, Houghton [127] reports ‘substantial net benefits in the longer term’ in terms of academic, societal and economic impact for ‘more open access’, due to increases in speed and breadth of access, as well as lower costs of publishing, driven by the move from print to electronic distribution [127]. A publisher’s revenue under gold OA depends on the number of accepted publications and the level of APCs, which might incentivize publishers to lower their acceptance criteria [75,240].^8^ This has given rise to the concerns about article quality discussed above, but also led to business models where OA mega-journals cross-subsidize more selective and thus less profitable journals from the same publisher [190].

### 3.2. Open/FAIR Data

Our review identified 67 studies which assessed the academic impact of Open Data [279–345]. Of these, 25 studies reported impacts on data reuse, 22 studies reported impacts on citations, 10 each on ethics/integrity and reproducibility, seven on efficiency and productivity, six on quality and equity, diversity and inclusion each, with further impacts on collaboration, and trust (two each).

Overall, few publications that are included in the review provide concrete evidence of impact of Open Data. Various publications study effects of data sharing policies, instead of effects of data sharing itself. The most obvious direct effect of data sharing is whether the data are actually re-used, and most literature focuses on this. By contrast to OA, if data are not available at all, they cannot be re-used, whereas research that is not OA can still be read and cited. Therefore, most studies do not compare usage statistics with a possible counterfactual. Another sizable part of the literature analyses whether making data openly available has some effect related to the publication that makes the data openly available itself, most notably, studying an ‘Open Data’ citation advantage, similar to OA. Other studies focus instead on whether studies that make data openly available increase the robustness of the results, related also to questions of reproducibility.

#### 3.2.1. Data reuse

In a wide variety of fields there are ongoing discussions of data sharing. A number of publications examine the use of datasets from existing repositories, such as in agriculture [339], biodiversity [105], ocean science [319], clinical trials [292,342], satellite imaging [302], environmental sciences [334], network sensing [306] and genomics [341]. Other publications introduce novel repositories, such as for topography [344], COVID-19 [333] or neurology [335,338] or at a specific university [308]. This does not represent an exhaustive list of repositories that are introduced, only publications that also report on actual usage.

Many papers discuss problems of data sharing, including challenges of common formats, standardization of vocabularies and ontologies [338]. Common standards also bring larger benefits for disciplinary specific repositories, such as the OpenNeuro repository [338] or the Genome Expression Omnibus repository [341], over general repositories, such as Zenodo, Dryad or Figshare. At the same time, Quarati & Raffaghelli [298], report that metadata quality is not associated with usage. Some argue for standards for citation of data [281], while others argue for also considering electronic supplementary material in considering data use [295].

Other papers discuss challenges in making data re-usable, as part of the findable, accessible, interoperable and re-usable (FAIR) definition [8]. Smale *et al*. [280] report a scarcity of published systematic evidence that data management plans (DMPs) have positive effects on data FAIRness. Bishop & Collier [303] found that researchers are influenced by ‘trusted brands’, such as well-known repositories, in trying to determine whether data will probably be re-usable or not. Bezuidenhout & Chakauya [305] report that reuse of data is constrained by limited resources in low/middle-income countries.

Qualitative data seems to be used most often for learning, not research, report Bishop & Kuula-Luumi [329], based on repositories in the UK and in Finland. Bishop [343] argues that sharing qualitative data is especially useful for teaching.

Several papers use theoretical models to explore the data sharing behaviour of researchers. Mueller-Langer & Andreoli-Versbach [320] found that authors may strategically delay publications to fully enjoy the benefits of studying the data for additional papers. Spiegelman [300] studied a model of data sharing in low- and high-quality science. They find that high-quality science is more likely to share data than low-quality science, and that data sharing can then be used as a marker of high-quality science.

#### 3.2.2. Open Data citation advantage

Piwowar & Vision [290] is the key study concerning the Open Data citation advantage, and is cited by most other articles for evidence of an Open Data citation advantage. They study gene expression microarray data and find that publications that share data openly receive 9% more citations than publications that did not share data openly, while controlling for a number of relevant confounders. This contrasts an earlier finding of Piwowar *et al*. [345] which found a much larger increase of 69%. Piwowar & Vision [290] find that 6% of the citations were made in the context of data reuse, suggesting that much of the citation benefit comes from additional citations due to data reuse. The exact mechanism is not clear, but the presence of some effect seems quite convincing.

Leitner *et al*. [289] also observe that publications which share data openly are cited more frequently, but they only control for publication year, and not for a range of other confounders, thereby offering little evidence of a causal effect. Drachen *et al*. [331] find evidence for a citation advantage of about 15% in astrophysics, which is possibly mostly caused by citations to datasets being shared. McGillivray *et al*. [297] report that special data papers have a positive impact on citations of the original research, suggesting that additional citations might indeed be associated with data reuse. Colavizza *et al*. [332] report about 25% higher citations for PLoS and BMC papers that link to data in a repository compared with papers that report that no data are available.

Zhang & Ma [313] studied the effect of the introduction of a data sharing policy at a journal using a difference-in-differences approach. They found that the Open Data policy increased citations by 1−4 times. The estimate of receiving four times more citations seems extremely large, and quite different from the more conservative estimate of 9% of Piwowar & Vision [290]. Huang *et al*. [321] performed a very similar study on the same journal, but reported an increase in citations of 1.5−2 times. Christensen *et al*. [282] also used a journal policy change in data sharing requirements, but reported no statistical differences between citations to articles shortly before and after the policy change. However, they did report almost double citation rates for articles that actually share data, although this might be due to some confounding factors.

Kwon & Motohashi [310] suggest that there are two competing effects of sharing data on the accrual of citations: a positive credit effect and a negative competition effect. While the credit effect leads to an increase in citations to publications that share data, it might lead to greater competition by other researchers, thereby crowding out citations to the original research. Both effects are reported to be present, where in the short term publications that make data available are cited more frequently, due to the credit effect, but in the long term, they are cited less frequently, due to the competition effect. Zhang & Ma [313] perform a similar analysis, and also find that that citation benefit is positive initially, but becomes negative later on.

A number of other articles also study citations to publications that either share or use Open Data. AlRyalat *et al*. [336] found that publications that used Open Data from BioLINCC receive a higher number of citations. However, they do not compare their results with other publications, making it unclear whether this is a causal effect. Coady *et al*. [342] report no significant differences between citations of publications using BioLINCC data and citations of other publications supported by the National Heart, Lung and Blood Institute, suggesting that there might not be an effect. Heller *et al*. [340] study conference proceedings in medical image computing (MICCAI) and find that papers that use open, public data are cited (or mentioned—formal citations were sometimes missing) 60% more frequently than papers using private data.

Raffaghelli & Manca [301] found that reading or citing of the data does not seem to be associated with the FAIRness of the data. Robinson-García *et al*. [284] study the Data Citation Index provided by Clarivate. They find that ‘data citation practices are far from common within the scientific community, with a high rate of uncitedness (88%)’ (p. 2970). They also find different data citation practices, with the natural sciences tending to cite datasets, while the social sciences and humanities tend to cite publications that introduce the data. Aleixandre-Benavent *et al*. [325] study open data policies and data availability across journals, and find no association with a journal’s impact factor. Piwowar & Chapman [326] on the other hand do report an association of data sharing with a journal’s impact factor, in addition to author experience. Neither study makes any causal claim.

#### 3.2.3. Reproducibility

One important reason for sharing data is that other researchers can then replicate the original research. However, research by Coady *et al*. [342] suggests that data are rarely re-used for this purpose.

Hardwicke *et al*. [288] studied whether the introduction of a data sharing policy at a journal affected the reproducibility of articles published in the journal. They reported that not all data appeared re-usable. Of the 35 articles for which data was re-usable, only 11 articles could be reproduced without author assistance, and an additional 11 could be reproduced with help. For the remaining 13 articles, at least one value could not be reproduced. Overall, 95% of the numbers produced in the articles could be reproduced (within a 10% error margin) using the open data, but it did take considerable time and effort. Hardwicke *et al*. [283] extended their previous study to articles with open data badges, and found similar results. Laurinavichyute *et al*. [330] also found that data sharing policies led to limited improvements in reproducibility. When code was shared, in addition to data, reproducibility greatly increased. Naudet *et al*. [291] analysed randomized controlled trials in BMJ and PLoS Medicine. Although only about half of the studies (17/37) shared data, most of those studies (14/17) could be reproduced but again required substantial effort and contact with the authors.

Nuijten *et al*. [314] compared papers and journals with data sharing (policies) with those without such data sharing (policies), and studied how they differ in terms of statistical inconsistencies. They found that data sharing (policies) do not affect statistical inconsistencies (as found through statcheck). Claesen *et al*. [293] corroborate this finding and also report no fewer inconsistencies when data are shared. Berberi & Roche [318] also report no association between data sharing mandates and article retractions or corrections, but this finding might suffer from selection bias [346].

One interesting issue raised by Thompson *et al*. [296] is that open datasets can be analysed multiple times, which, coupled with publication bias, may result in increases in false positives in the literature. Besides some potential technical fixes within the realm of null hypothesis testing, the authors suggest three alternatives: Bayesian analysis, predictive cross-validation with held-out data, or most radically, considering all research on open data as exploratory. Drewry *et al*. [347] discuss the benefits of Open Science practices in pharmaceutical research, such as SGC laboratories’ open sharing of chemogenomic data. They highlight enhanced collaboration, increased reproducibility and faster scientific progress as significant benefits.

#### 3.2.4. Efficiency/productivity

Cannon *et al*. [309] found no effects of a new data sharing policy on submission rates, acceptance rates and peer-review times. Holt *et al*. [312] did find that the introduction of a data sharing policy increased the time editorial staff spent on processing a manuscript, which was mitigated after some modifications to the workflow. Grant & Hrynaszkiewicz [337] found that simpler data availability statements required less additional editorial time than data availability statements that pointed to public data.

#### 3.2.5. Ethics and equity of data sharing

Cummings *et al*. [307] studied whether Open Data sharing makes a difference to participants consenting to take part in research, but found no such influence. They do raise one issue, namely that participants’ consent is usually only valid for the original study, while data sharing provisions allow the reuse of data for other purposes, without acquiring consent from the original participants, a point also made by Bishop [343] and Mozersky *et al*. [279] in the context of qualitative data. Although researchers often raise concerns of privacy and ethics in the context of qualitative data, research participants may be comfortable sharing for instance interview transcripts [316]. Nonetheless, there might also be challenges and ethical concerns in secondary analyses of qualitative data as these data are situated in their context, although with care, it might be possible to integrate and analyse qualitative data across projects [327], and in some fields, such as health science, the situatedness may be less salient in the research [279].

Xia & Liu [341] report a divide in the use of genomic data between the Global North and the Global South, with essentially none of the data being used by Latin America and Africa, indicating that benefits of shared data mainly accrue for the Global North.

Abebe *et al*. [317] argue that those whose data is being shared might not enjoy the benefits, and that a deeper understanding of, and engagement with, the local context is necessary to make data practices more equitable. A similar argument is made by Carroll *et al*. [311] who explored the intersection between data and tribal rights of indigenous people, arguing that indigenous people should regain sovereignty and governance over their data. Carroll *et al*. [324] argue for integrating the CARE Principles for Indigenous Data Governance (Collective Benefit, Authority to Control, Responsibility, and Ethics), in addition to the FAIR principles, when considering open data. Khalil *et al*. [304] plead for considering adopting ethical views on open science development to avoid potential issues when implementing open science.

Bezuidenhout *et al*. [285] report that access to data alone is not enough to make effective use of it. They highlight that ‘an emphasis on access fails to capture the social and material conditions under which data can be made usable, and the multiplicity of conversion factors required for researchers to engage with data’ (p. 473). Moreover, similar barriers may affect data sharing: ‘The absence of pre-publication data and the slow rate of academic publications diminish the impact that LMIC [low- and middle-income countries] research has in the global research community. Nonetheless, it is precisely because of the slow rate of publication that LMIC scientists are hesitant to share pre-publication data with anyone with whom they do not have a personal connection’ [305, p. 47]. Using an innovative approach, Shanahan & Bezuidenhout [328] report that online material is also not equally accessible around the world, not only due to connectivity issues such as low bandwidth, but also due to explicit geoblocking and restricted access.

### 3.3. Open Methods

We identified 10 studies related to Open Methods [348–357]. Three studies investigated effects of Open Methods on methodological *quality*. Bakker *et al*. [348] investigated whether requiring explicit pre-data collection power analyses increased statistical power in reported results. The intervention seemed ineffective overall, however, with no difference in the planned sample sizes stated. In a related study, Bakker *et al*. [351] examined the impact of pre-registrations that were either structured (‘with detailed instructions’) or unstructured (‘minimal direct guidance’) to find that structured pre-registrations were more effective in reducing deviations in published studies. Studying the effects of registered reports on adherence to OS practices in psychological research, Susanin *et al*. [355] found an increase in ‘the use of data sharing, reporting of statistical form, and inclusion of start-stop rules’.

Next, related to *quality* and *efficiency*, Hocker *et al*. [357] investigated the use of an open tool for reuse of coding schemas for qualitative research, finding the tool improved both time taken to complete the task as well as coding accuracy.

Regarding *quality* and *citations*, Markowitz *et al*. [354] observed no effect of OS practices in communication research on citation rates or the prevalence of *p*-values just below *p* < 0.05, but they did note an increase in non-significant *p*-values, suggesting a potential impact on transparency and honesty.

Relating to *citations*, Schafmeister [352] studied whether publication of independent replications affected levels of citedness of original studies. They found that successful replication was associated with around a 5% increase, and unsuccessful replication with a 4% decrease in citations.

Finally, regarding *reproducibility*, Ebersole *et al*. [349] found that review of protocols for replication studies in advance of replication attempts had ‘little impact’ on levels of replication.

### 3.4. Open Code

Nine studies were identified addressing this theme [358–366], five relating to efficiency gains in the production of research-enabling software via open source community efforts [367], three relating to a citation advantage for publications with Open Research Software [368] and two examining effects of Open Code on computational reproducibility.

Regarding *efficiency*, Ratib *et al*. [365] found that open source enabled faster development of tools that are better adapted to user needs (via users contributing to development, and larger numbers involved in development and testing). Blasco *et al*. [358] found that use of open data and algorithm development competitions in computational biology and bioinformatics leads to better performing algorithms, especially through quick ‘exploration of the solution space’ and creation of ensemble techniques through collaboration. Coetzee *et al*. [359] reviewed open source software and open data initiatives in the geospatial domain, concluding that open source geospatial software and open geospatial data have changed data collection (including via crowdsourcing), processing, analysis and visualization. Similarly, Ratib & Rosset [362], in their case study on the OsiriX Biomedicine tool, reported that its open source nature and embedding in a very responsive community equated to ‘software updates and new features at a rate that exceeded by far the rate of software updates in the industry’ (note that the authors do not quantify this claim, however). McCormick *et al*. [361] describe the open source ITK (Insight Toolkit) software, a ‘reproducibility verification infrastructure’ with community code review. They report efficiency gains (operationalized as fewer ‘fix-up commits’). Finally, Wallace *et al*. [363] report on development of RT-Cloud, an open source cloud-based Python software package for real-time functional magnetic resonance imaging (fMRI) experiments, finding efficiency gains in terms of ease of set-up/maintenance, reduced costs and ease of scaling.

Regarding impact on *citations*, Heumüller *et al*. [360] studied papers from Software Engineering conference and found a slight citation advantage for papers which had made software artefacts available over those which had not. However, Winter *et al*. [364] also studied levels of citations of articles with associated Open Code and found no general effect of Open Code on citations.

Finally, Schröder *et al*. [366] assessed the computational *reproducibility* of Jupyter notebooks associated with journal publications. They found that just 3 of 22 notebooks could be reproduced (i.e. ‘not only all cells run successfully but also the output is equal to the originally published version’), suggesting the need for improvements in code quality.

### 3.5. Citizen Science

We identified 129 studies as relevant to Citizen Science (CS) [43,369–496]. More than two-thirds (89 studies) of these report on the impact of CS projects in ecology or environmental research, and especially in activities to monitor flora and fauna. The main types of academic impacts of employing CS in these monitoring activities relate to efficiency in data collection, as well as concerns over data quality. Academic impacts demonstrated by a range of studies are the increased temporal and spatial scales of monitoring activities enabled by CS methods, coupled with moderate to substantial cost savings. Many state that CS can be a useful complement to existing approaches [393,410,420].

A key question is which specific mechanisms drive the various forms of academic impact attributed to CS. While there are certain challenges related to organizing large groups of volunteers, it is not surprising that more people will be able to conduct monitoring activities on larger temporal and spatial scales. It is thus clear that there exist trade-offs ‘between cost-effectiveness and involvement (i.e. societal impact), as well as between data richness and data quality’ [463]. Besides the impacts on efficiency and quality in data collection, CS has also been suggested to lead to novel insights and challenge existing approaches, due to the diversity of backgrounds, perspectives and skills of citizens [399,404,426,429,465]. At the same time, CS activities seem to be concentrated in the Global North, limiting the global diversity [375,411,412,414,423,455]. Some researchers have raised ethical questions around how to properly credit CS volunteers [375,403,414].

#### 3.5.1. Data quality

Earlier literature, such as the review by Dickinson *et al*. [402], reported quality issues with citizen-generated data. More recent reviews generally agree that CS projects can generate data of sufficient quality for monitoring activities [383,412,477]. As shown below in table 4, most studies we examined (46 studies) report that the quality of data gathered through CS is of the same, if not higher, as data gathered by professionals. However, as Aceves-Bueno *et al*. [477] make clear, there is not a single agreed upon definition of when data are deemed ‘reliable’ or of ‘sufficient quality’. Indeed, eight studies also report worse data quality and 24 report neutral or mixed impacts on quality, and data might be good enough for some purposes, but insufficient for others.

**Table 4.** Overview of CS studies relevant to ‘quality’, and whether outcomes were positive, neutral/mixed or negative.

type of change	relevant studies
positive	Aceves-Bueno *et al*. [401]; Agostini *et al*. [416]; Aplin *et al*. [370]; Barrows *et al*. [492]; Biraghi *et al*. [410]; Branchini *et al*. [490]; Brown *et al*. [389]; Cruickshank *et al*. [468]; Crumley *et al*. [390]; Dallaqua *et al*. [446]; Danielsen *et al*. [460]; Delaney *et al*. [456]; Dodson *et al*. [436]; Facchinelli *et al*. [443]; Farhadinia *et al*. [407]; Fehri *et al*. [431]; Fehri *et al*. [475]; Flesch and Belt [419]; Fowler *et al*. [371]; Fuccillo *et al*. [386]; Hochmair *et al*. [440]; Jones *et al*. [459]; Kasten *et al*. [464]; Keeping *et al*. [395]; Lawson *et al*. [437]; Leocadio *et al*. [432]; Lin *et al*. [489]; Lovell *et al*. [380]; Manda *et al*. [384]; McKinley *et al*. [405]; Meentemeyer *et al*. [409]; Meschini *et al*. [470]; Njue *et al*. [412]; Petrovan *et al*. [491]; Porter *et al*. [466]; Pusceddu *et al*. [494]; Quinlivan *et al*. [383]; Shumba *et al*. [379]; Soroye *et al*. [462]; Starkey *et al*. [434]; Swanson *et al*. [373]; Van Der Velde *et al*. [422]; Van Emmerik *et al*. [425]; Vianna *et al*. [377]; Watson and Floridi [427]; Weigelhofer, Pölz, and Hein [415]
neutral/mixed	Beck *et al*. [429]; Bison *et al*. [421]; Buytaert *et al*. [413]; Cox *et al*. [442]; Crall *et al*. [387]; DiBattista *et al*. [418]; Dickinson *et al*. [402]; Engel and Voshell [495]; English *et al*.s [447]; Foster-Smith and Evans [485]; Gardiner *et al*. [452]; Giovos *et al*. [417]; Gunko *et al*. [449]; Harvey *et al*. [420]; Kallimanis *et al*. [467]; Kremen *et al*. [441]; Meixner *et al*. [469]; Périquet *et al*. [476]; Ratnieks *et al*. [433]; Shin *et al*. [398]; Soroye *et al*. [496]; Swan [426]; Thel *et al*. [392]; Tran *et al*. [439]
negative	Alabri and Hunter [438]; Galloway *et al*. [484]; Johansson *et al*. [394]; Robinson *et al*. [391]; Roy *et al*. [445]; Smale *et al*. [473]; Theobald *et al*. [448]; Yang *et al*. [388]

Many CS studies focus on biology/ecology, and problems of data quality often relate to less accurate identification of species, or over- and under-reporting of the abundance of certain species. In some instances, more common, or more ‘likeable’ species are more likely to be reported (e.g. [468,476]). In other cases, CS seemed to be biased towards over-reporting rare species (e.g. [398,417,484]), possibly due to the greater ‘surprise’ of observing such a species. Other rare events might also be more likely to be observed by CS [434,443]. The results might vary according to which people are involved in data collection [430].

Aceves-Bueno *et al*. [477] suggest that studies might be overstating the accuracy of CS, because many studies have been explicitly designed to test the accuracy. Moreover, it is possible that positive outcomes are more likely to be reported in the literature; Theobald *et al*. [448] report that only 12% of the surveyed projects have contributed data to peer-reviewed articles (see also Conrad & Hilchey [374]). In addition, these projects are more likely to have invested a lot of effort in training and gathering good data. Indeed, training [464] and protocols [495] in data collection seem to be important determinants of data quality [384,388,411,433,474]. On the other hand, the literature may underestimate the accuracy of CS, because many studies ignore uncertainty, or potential errors, in professionally gathered data [477]. As several others also argue [378,482,483], the study design might be more relevant to data quality than the use of CS *per se*.

#### 3.5.2. Efficiency

Sauermann & Franzoni [424] estimate the average contributions of volunteers to represent a value of about $200 000 during the first half year of a project. Generally, CS was found to have great cost savings compared with traditional surveying for monitoring [378,407,452,454,460] or experiments [415,493]. Most studies report higher start-up costs, but lower running costs, so that longer studies [401] or larger studies [453] could be more efficient. Indeed, CS was found to be most effective at large spatial and temporal scales [411,443,465,478]. Combining traditional and CS monitoring might be more effective [434,444], and combining CS with AI possibly even more so [385].

### 3.6. Open Evaluation

We identified 16 articles relating to Open Evaluation [14,497–511], i.e. open peer review, including disclosure of reviewer identities (Open Identities) and publication of review reports (Open Reports). We here report those findings under the themes of effects on quality (eight studies), ethics and integrity (five studies), efficiency and productivity (five studies), citations (three studies) and equity, diversity and inclusion (two studies).

#### 3.6.1. Quality

Eight articles investigated effects on review quality of models of Open Identities. In Bianchi & Squazzoni’s [497] theoretical model simulating competition and status dynamics in peer review, Open Identities led to reductions in quality of processes under conditions of high levels of status awareness and competition among reviewers. Such results are conceded by the authors, however, to be ‘only abstract and highly hypothetical’. In fact, empirical investigations of effects of Open Peer Review on review quality seem to generally show neutral to positive effects.

Two studies found neutral impact of Open Identities on quality. Van Rooyen *et al*. [502] conducted a randomized trial at a medical journal, where manuscripts were evaluated under either Open Identities or standard (anonymized) review. No significant differences in review quality were found. A similar randomized control trial (RCT), also at a medical journal [509] found no difference in review quality.

Three studies found a positive correlation between Open Identities and indicators of quality, however. Walsh *et al*. [507] performed an RCT at one psychiatry journal with reviews performed under Open Identities found to be of significantly higher quality than those conducted under blinded review. Thelwall [506] analysed open review reports from MDPI journals, where Open Identities is optional, finding that signed reviews were on average 15% longer (although with no difference in publication recommendations). Thelwall speculated that increased review length is associated with review quality. Fox’s [511] study of optional Open Identities at one ecology journal similarly found that signed reviews tended to be longer. However, interpreting these latter two findings requires caution. When optional, it is possible (perhaps likely) that reviewers are simply less likely to sign reviews they perceive themselves to be of lower quality (measured here by the questionable proxy of review length).

Finally, two studies looked at effects of Open Identities and Open Reports in combination upon review quality. Kowalczuk *et al*. [500] performed a comparative assessment of peer review reports employing open or closed review models at three otherwise comparable medical journals (BMC Infectious Diseases—Open Identities and Open Reports; BMC Microbiology—single-blind; Journal of Inflammation—both models). Reviews at the journal employing open peer review were found to be of 5% higher quality, although at Journal of Inflammation there was no difference in quality in reviews under different models. Finally, Bornmann & Daniel [508] studied levels of inter-reviewer agreement in the journal Atmospheric Chemistry and Physics, which uses Open Identities and Open Reports. They found similarly low levels of agreement to that reported in other studies of reviews conducted under closed review models.

#### 3.6.2. Integrity

Open Identities’ effects upon the integrity of review processes and reviewer behaviour seem complex. Wolfram *et al*. [14] studied how journal policies for Open Identities (optional or mandatory) impacted reviewers’ use of ‘hedging’ (terms indicating lack of certainty) and ‘research-related terms’ when evaluating manuscripts, finding that opening identities made ‘no appreciable difference’ and concluding that ‘reviewers are not influenced by the identification requirements’.

Other studies did find differences, however. Regarding tone, courtesy and constructiveness of comments, Walsh *et al*.’s [507] RCT study of optional Open Identities found that where reviews were signed, they tended to be more ‘courteous’. Felizardo *et al*. [499] conducted a small survey (12 reviewers, six authors from Software Engineering) to understand how respondents believed their review behaviour changed when not anonymous. The majority reported being more likely to write ‘bland and cautious’ reviews that avoid issues of novelty/general interest and focus on more ‘objective’ issues like technical concerns. Matsui *et al*. [501] used sentiment analysis of review reports to assess (among other aims) how reviewer disclosure of identities (published optionally online after single-blind process) is associated with review sentiment, finding that reviews written by reviewers who choose to ‘sign’ were ‘more subjective and more positive than the anonymous reviewers’ reviews’. The authors contend this implies ‘possible social pressure from name association’, although since authors chose to sign, we might more readily posit that authors were more inclined to sign when reviewing positively. Finally, regarding effects from both revealing reviewer identities and publishing reports, Bravo *et al*. [498] found that male reviewers tended to be more ‘constructive’ under these conditions.

#### 3.6.3. Efficiency

The aforementioned simulation work of Bianchi & Squazzoni [497] suggested that efficiency could be negatively affected in terms of reviewer time. Similarly, the simulation study of Radzvilas *et al*. [505] suggested that Open Identities could ‘increase the effort of authors in a range of circumstances’, but this effect is influenced by multiple factors including reviewers’ own levels of effort. Supporting these model-driven findings, Walsh *et al*. [507] and Van Rooyen *et al*. [502] found that under Open Identities, signed reviews took longer to complete than those not signed. Finally, Bravo *et al*. [498] found that open reports and optional open identities had no significant change on referees’ willingness to review or time taken to review.

#### 3.6.4. Citations

Regarding effects on citations, Zong *et al*. [503] conducted a scientometric study using data from the *PeerJ* journal, where revealing identities and publishing reports are optional, to find that ‘articles with open peer review history could be expected to have significantly greater citation counts than articles with closed peer review history’. Wei *et al*. [510] also studied effects of open peer review (OPR) on article citations at six PLoS journals, comparing reports under open to those under closed review. They found that articles reviewed under open conditions were associated with higher average page views, saving and sharing, but found mixed effects upon citations, with between 6.2% and 7.5% more citations in Google Scholar, Dimensions and Semantic Scholar, but no significant association between OPR and citations in the more selective Scopus and Web of Science databases (hence the boost in citations may be attributable to higher citations of openly reviewed articles in ‘grey literature’ found in Google Scholar, Dimensions and Semantic Scholar but not Scopus or WoS).

Regarding reviewer requests for self-citations, Levis *et al*. [504] compared prevalence of citation requests for reviewers’ own work in open and closed conditions, controlling for overall number of citation requests. They found that ‘in reasonably similar journals that use single-blind and open review, there were no substantive differences in the pattern of peer reviewer self-citations’.

#### 3.6.5. Equity, diversity and inclusion

Finally, two studies reported findings applicable to equity, diversity and inclusion. Firstly, Bianchi & Squazzoni’s [497] simulated model suggested that perceived status of open reviewers impacts reciprocity strategies. If so, they advise that ‘transparency can be especially detrimental in cases of young scholars who could be sensitive to the risk of retaliation when asked to review work by more advanced senior scholars’. Fox [511] found that male reviewers were 1.8 times more likely to sign reviews than female reviewers, which may have negative consequences for reviewer recruitment and the ‘diversity of reviewers recruited by journals’.

### 3.7. Open Science in general

In this section, we cover studies that demonstrated academic impacts but did not fit pre-specified categories, addressing multiple types of Open Science (OS) or a broader perspective on OS. Our review identified 21 studies from general Open Science [28,347,512–530], showing impacts (or lack thereof) on diverse areas such as reuse (eight), efficiency and productivity (six), quality, (six), citations (five), collaboration (five), equity, diversity and inclusion (four), ethics and integrity (three), reproducibility (three), trust (three) and others (two).

McKiernan *et al*. [522] reviewed the prior literature showing ‘open research is associated with increases in citations, media attention, potential collaborators, job opportunities, and funding opportunities’.

An emerging approach to signify the application of OS practices is to award ‘badges’ to journals or articles. These badges display different types of adherence to OS practices, such as Open Data and Open Code. Schneider *et al*. [526] observed a positive effect of badges on trust in results among scientists and students, but not the general public, possibly due to the public already assuming openness. However, Schneider *et al*. [526] also noted that this effect might not be generalizable without additional research. Zong *et al*. [529] reported that while Open Science badges increased social media attention, they did not affect citation counts.

Research on OS impacts often appears as case studies. Anagnostou *et al*. [512] highlighted a platform in Ghana based on open repositories and digital research tools for ageing research, enabling local insights for research and policy. Investigating the case of ‘blue economy’ (sustainable use of ocean resources for economic growth), Coro [517] described how OS practices improved computational process speed, collaboration, interdisciplinarity, virtual laboratories, model reuse, data longevity and findings dissemination.

Other case studies address efficiency and equity in research. A review covering four case studies from Argentina [513] suggests that OS practices support ‘efficiency, democratization and social responsiveness’. The authors also note ‘several directions of openness could lead to different benefits’, implying that partial openness can also be beneficial.

On the other hand, Besançon *et al*. [515] highlight how the urgency of the COVID-19 pandemic led to suboptimal adoption of OS practices (especially Open/FAIR Data, Open Code and Open Peer Review) and (in some cases) misuse of preprinting slowed the response of the academic community. On the same topic, Tse *et al*. [530] argue that Open Data, by sharing protein structures, and Open Source practices, such as sharing information on potential inhibitors to COVID-19, have greatly contributed to accelerating research into COVID-19.

OS may also create negative effects. Hofmann [519] and Ross-Hellauer *et al*. [28] warned of risks like reduced quality, inclusiveness and reinforced gender divides, along with reliance on specific skills and infrastructure. These effects are difficult to quantify, but awareness is crucial for understanding OS impact pathways.

Lastly, LeBel *et al*. [521] scrutinized concerns about OS practice costs, such as increased false negatives and opportunity costs associated with larger samples and direct replications. They argue these concerns are unwarranted, as OS aims to reduce Type I error rates and increase statistical power. Despite short-term challenges, the benefits of open and high-powered research outweigh the costs in the long run.

In conclusion, while Open Science practices show benefits across various fields, they also present potential challenges and risks that need careful management. Further studies and refined models of impact pathways are necessary to fully understand and optimize these practices in diverse research contexts.

## 4. Discussion

Our scoping review shows that there is a substantial body of literature (485 studies) demonstrating academic impacts of OS practices (RQ1). These studies covered all OS practices (Open Access—OA, Citizen Science—CS, Open/FAIR Data—OFD the most addressed practices), and effects on various aspects of impact (most common were effects on citations, quality, and efficiency and productivity) (see figure 2).

Regarding **SRQ1** (types of impact, including whether positive/negative), table 5 below presents an overview of our findings. We found a range of **positive impacts**, most notably effects of OA upon levels of *citations,* although importantly, effects vary by OA type and differ across disciplines. We further found moderate to neutral effects of open peer review upon review *quality*, *efficiency* gains due to cost savings in Citizen Science (ecological monitoring), OA (publishing costs, speed of dissemination due to preprints) and general gains in effectiveness of research in response to the COVID-19 pandemic. We also found positive impacts on *reuse* of Open/FAIR data, *reproducibility* (due to pre-registration), as well as some limited evidence of positive effects on *novelty, collaboration* and *trust*. **No or mixed impacts** included effects upon *quality* of articles due to OA and data from CS, *efficiency* of editorial processes due to OFD and Open Peer Review, *reproducibility* (OFD not associated with levels of statistical consistency, but still associated with high reproduction costs; Open Methods of pre-registration or protocol-sharing not effective in increasing sample-sizes or reducing *p*-values just below 0.05, but increasing levels of reporting of non-significant *p*-values), and *integrity* in open peer review processes. **Negative impacts** included those on *equity/diversity/inclusion* (APC barrier and potential for reuse of OFD barriers for less well-resourced researchers, as well as negative effects on inclusivity and gender equity), *efficiency* in OA (time lost due to engagement with predatory publishing), as well as *ethics* due to potential for re-identifying participants and data colonialism (lack of benefit-sharing) in OFD. These effects are most commonly directly contributable to OS practices.

**Table 5.** Summary of key findings. Findings based on individual studies are highlighted with an asterisk (*). Abbreviations: Open Access (OA), Open/FAIR Data (OFD), Open Methods (OM), Open Code (OC), Citizen Science (CS), Open Evaluation (OE), Open Science in general (OSG).

	positive impact	no or mixed impact	negative impact
**citations**	OA: small to moderate increase for OA papers, although with differences across OA types (findings of a citation advantage less common for gold OA compared with hybrid and/or green OA); significant increase for publications which were earlier available as preprints. OFD: small to moderate increase. OE: neutral to moderate effect of OPR.	OC: unclear effects. OSG: positive impact of OS badges on social media attention (tweets) but not citations.	
**quality**	OE: open peer review has moderately positive to neutral effects on review quality.	OA: conflicting evidence as to the effect of OA on article quality. Preprints found to be of same or similar quality as published versions. CS: given adequate training, data quality often comparable with other sources, although some studies report negative effects.	
**efficiency and productivity**	CS: cost savings in ecological monitoring; expanding spatial and temporal scales of data collection. OA: lower publishing costs; significant increase in speed of dissemination due to preprints. OSG: OS contributed to speed and scope of research activities during COVID-19 pandemic response.	OFD: no substantial effect of current data sharing policies on editorial effort.* OE: unclear impact on overall efficiency, but tendency towards slightly longer review times under open identities. OA: mixed effect on number of published articles per journal.	OA: wasted time (esp. among junior researchers) engaging with predatory publishers.*
**equity, diversity, inclusion**	OA: research published via OA involves more (international) collaboration*; OA publications receive more diverse citations, suggesting more universal use of research.*		OA: APC model has effects of stratification upon patterns of authorship, tending to marginalize those with fewer resources.
			OE: gender and status dynamics regarding open identities (male, more senior reviewers more likely to adopt).* OFD: reuse of data more challenging for those with fewer resources. CS: CS activities tend to be focused in the Global North.
**reuse**	OFD: positive effect on reuse, but largely constrained to well-resourced researchers.		
**reproducibility**	OM: structured preregistrations decrease deviations from protocol*; positive effect of registered reports on Open Science practices related to sound statistical practice.*	OFD: data sharing policies have no clear effect on statistical inconsistencies*; high costs for reproduction even though data shared. OM: no impact of requiring sample size justification on sample size*; no impact of reviews of protocols for replication studies on replicability of findings*, no effect of Open Science practices on citations or *p*-values just below *p* < 0.05, but increase in reporting of non-significant *p*-values.*	
**novelty**	OSG: positive effect of OS practices on rate and frequency of true discoveries, despite short-term costs.*		
**ethics and integrity**		OE: unclear impact of open reports and open identities on integrity of review process: some find no impact, some do. OE: no effect of OPR on reviewer requests for self-citation.*	OFD: making data public might involve risk of re-identifying participants.* OFD: whose data is being shared might not enjoy the benefits.
**trust**	OSG: positive impact of OS badges on trust in results by scientists.*		

In terms of specific mechanisms responsible for the impacts of OS (**SRQ2**), we found similarities, but also important differences, among OS practices. Impacts of OA and OFD on citations and reuse work in similar ways: open publications/data → higher readership/reuse → increased citations. This mechanism is only part of the picture, as there are multiple moderating factors (see below). For CS, the main mechanism of impact is reduced cost of data collection (efficiency), which leads to the ability to conduct data collection across increased temporal and spatial scales (effectiveness). For Open Methods (OM), we did not find many impacts, but where we did, they are driven by increased guidance and structure in terms of research methodology. In the case of Open Evaluation (OE), increased accountability due to open reports or identities leads to neutral to moderate effects on quality of reviews, but may lower efficiency with some studies finding that reviews take longer to complete (although others find no effect).

As stated, the implied mechanisms involve certain enabling and inhibiting factors that moderate the impact of OS (**SRQ3**). An important inhibiting factor is the requirement for potential beneficiaries of open resources to have sufficient capacity in terms of skills, resources and infrastructure. Examples are of the OA APC-barrier, which presents a financial obstacle that influences who is able to publish in which journals (disadvantaging researchers from less resourced institutions and the Global South more broadly) and reuse of OFD which often requires sufficient skills, equipment and infrastructure, lack of which at least partly explains the low reuse of data by researchers in the Global South. While skills and resources can act as barriers to the impact of OS, the reverse is also true: our review found sufficient training of volunteers in CS projects to be a key factor in ensuring sufficient data quality. Similarly, the existence of funding and institutional support enables researchers to overcome potential efficiency costs of OM more broadly (including sharing of data and code, preregistration and similar practices). Lastly, epistemic diversity (different approaches to generating knowledge) also acts as a factor moderating the impact of OS. For example, quantitative approaches might benefit more directly from shared data than qualitative methods, while missing context of anonymized qualitative data inhibits further reuse.

Although our review identified a variety of academic impacts stemming from a range of OS practices, substantial knowledge gaps remain (**SRQ4**). We found, for instance, relatively few studies addressing efficiency gains or losses due to OS workflows (e.g. data or code sharing), the efficacy of DMPs to promote higher quality data sharing, or to which extent Open Methods directly improve quality and reproducibility of research.

A salient issue that posed a challenge in conducting our review is that many studies we encountered report on the *uptake* of OS, rather than OS’s *impact*. This is not problematic *per se*, but becomes an issue when policy is built on assumptions about impacts deriving from uptake, where the *impact* itself has not been rigorously studied. In addition, from the overview gained via this process, we note that many studies which were initially thought potentially relevant were eventually excluded for their lack of empirical observations, presenting arguments rather than evidence.

Much of the research summarized in our study comes via case studies or is based on small samples (e.g. individual data repositories or Citizen Science projects). Findings from case studies can be difficult to generalize. Although such case reports provide evidence of the potential impact of OS, it may also bias the evidence. projects that failed to set up a successful data repository, or failed to finish a Citizen Science project, may be less likely to report on it. In addition, we note that many of the authors of such reports are involved in the projects themselves, potentially bringing other forms of bias or conflicts-of-interest. For these reasons, more independent research on OS is necessary.

One set of challenges in studying the effects of OS on academic impact is methodological. At a broader level of impact assessment, studies often rely on post hoc investigations of existing datasets, where establishing a credible counterfactual is often difficult. For example, while it is quite straightforward to compare citations of OA and closed access, it is much harder to compare reuse of Open Data versus closed data, since closed data that has been re-used is hard to identify. Moreover, the central problem in this challenge is distinguishing impacts *of* Open Science from the effect of Open Science *on* impact. That is, Open Science may have some impact, but it might not immediately be clear whether this impact depends on the fact that it was ‘open’. For example, consider software that is developed by researchers. Suppose it is released as Open Source. The software might be used by thousands of researchers, and hence, it could be considered to have academic impact. However, it is possible that the exact same software might have also been used by thousands of researchers had it been licensed by a commercial company. If that were the case, whether the software was open or closed would have had no effect on the overall use by researchers, and there would be no effect of Open Science on academic impact.

At the same time, many studies are not able to disentangle effects of OS from other potentially confounding factors (such as selection bias or journal prestige in the case of the OA citation advantage), thus challenging claims of causal effects. Many studies lacked consideration of causality, simply reporting overall figures. Some studies do compare ‘Open’ outputs with ‘Closed’ outputs, but are not explicit about causality, even if some of the conclusions are being drawn and stated in causal terms. This is a particular issue in much of the citation impact literature considered here, where despite the large number of studies investigating the OACA, we note the lack of a consolidated understanding of causal factors affecting citations. In addition, the literature on OA and OFD employs a large variety of bibliometric methods, some of which are inadequate to answer the posed questions. We therefore echo the call for reporting guidelines for bibliometric studies by Langham-Putrow *et al*. [150].

A small minority of publications explicitly considers causality, and only a few of them (including some randomized controlled trials) are able to make a strong case for causality. Drawing causal conclusions is indeed something that is very challenging, and sometimes simply impossible. Although we call for greater attention on causality in studies on Open Science, it is even more important to consider the limitations when interpreting studies without a clear causal understanding, and draw conclusions and provide policy advice that is commensurate with the (lack of causal) evidence [346].

Beyond methodological issues around evidence of academic impact of OS, we must be careful in appraising the available evidence, keeping in mind that what has been researched most might not be the most relevant aspects of OS impact. For instance, much of the literature focuses on citations, and the citation advantage, be it of Open Access, Open Data or Open Code. This is a clear instance of a streetlight effect: it is easy to measure and study, even if drawing causal conclusions remains challenging. This is not to say that citations are irrelevant; they do provide some insight as to the increased (and potentially broader, cf. [182]) use of OS. But there are other academic impacts, such as quality, reproducibility, efficiency and equity, that merit much more attention, even if they are more difficult to study. As stated by UNESCO, OS should be about ‘making science more accessible, inclusive and equitable for the benefit of all’ [531]. The research community should ensure that we study the impacts that are relevant in achieving that.

## 5. Conclusion

Our scoping review demonstrates a range of academic impacts of Open Science, both positive and negative. The majority of reviewed studies investigated effects of Open Access, Citizen Science and Open/FAIR Data, with fewer studies reporting effects of Open Evaluation, Open Code and Open Methods. Key areas of impact studied are citations, quality, efficiency, equity, reuse, ethics and reproducibility, with most studies reporting positive or at least mixed impacts. However, we also identified significant unintended negative impacts, especially those regarding equity, diversity and inclusion. Overall, the main barrier to academic impact of OS is lack of skills, resources and infrastructure to effectively re-use and build on existing research.

This scoping review has several limitations. The inclusion only of English-language studies and use of a limited number of cross-disciplinary databases for initial search (Web of Science and Scopus) may have led to the exclusion of relevant studies. Web of Science and Scopus are known to have issues of representation regarding disciplines (i.e. Social Sciences and Humanities) and regions (i.e. Global South) [532–535]. These issues are probably somewhat mitigated by our use of OpenAlex, which has much broader coverage [536,537], for the second search phase, however. Relatedly, pilot searching indicated the large numbers of results returned by key terms. Hence, our search strings were somewhat restricted to balance results retrieved and pragmatic concerns for resource constraints. This means some key terms for OS practices, like preprints, preregistration or open hardware, were not included. This may have excluded relevant studies that used narrower or less common terms in their titles or abstracts. Some omissions (e.g. preprints) were specifically targeted during the snowballing phase to somewhat make up for any such limitations. These limitations could help explain our inclusion of only nine references each for Open Methods and Open Code, which may have been mitigated by inclusion of keywords like protocols, research software, open source software and academic software (our thanks to one reviewer for pointing this out). Further, because of our limitations on terms to include in search strings, and our updating of categorizations of academic impacts as we analysed the literature, our search terms excluded specific mention of some aspects reported here (ethics, diversity, inclusion, reproducibility, novelty, reproducibility) meaning some relevant studies related to those aspects may have been missed in our initial search. Finally, our screening approach involved single screening at certain stages. Despite regular check-ups and feedback within the team to strengthen inter-coder reliability, this nonetheless may impact coding reliability.

While it is not our goal to provide specific recommendations for stakeholders, we can nonetheless draw some conclusions regarding implications for future research and policy. First, despite the wealth of studies reviewed, important knowledge gaps around the efficacy of Open Methods and DMPs to improve reproducibility, and the effect of OM on efficiency remain and should be addressed. Second, independent investigations across initiatives, instead of individual case studies of successful initiatives, would be better equipped to build a systematic evidence base of the academic impacts of Open Science. In methodological terms, monitoring OS should expand from a focus on *uptake* towards impact. In extension, monitoring of impact must go beyond what is easy to measure or where data sources are readily available, which could be achieved with increased funding of research infrastructure for meta-research and monitoring efforts. Finally, more systematic agreement on standards/guidelines for reporting of bibliometric studies, as well as a greater focus on causality and limitations of correlational approaches would aid in establishing rigorous evidence for the academic impact of OS.

## Data Availability

Datasets and additional materials supporting this article are published on Zenodo [42]. Supplementary material is available online [538].

## Footnotes

^1^
Complementary reviews by our collaborators separately tackle *societal* [38] and *economic* (forthcoming) impacts.

^2^

https://unpaywall.org/

^3^
We hence do not include literature which reports only on the uptake of or attitudes towards Open Science and its various aspects.

^4^
We excluded Open Government Data from this study because we focused on OS practices within academia.

^5^
We excluded literature on crowdsourcing. Although some papers refer to a crowdsourcing approach as Citizen Science [43], approaches to crowdsourcing might also involve paying (online) workers, such as using Amazon Mechanical Turk. For this reason, we considered crowdsourcing out of scope for our review.

^6^
In addition, ‘diamond’ OA more recently refers to OA journals with no author facing charges, which can be understood as a subset of gold OA. Only one study identified in our review analysed diamond OA journals.

^7^
Closed-access journals that offer the option to publish manuscripts OA under an open licence by paying an article processing charge (APC) are commonly referred to as ‘hybrid’.

^8^
Note, however, that many OA journals listed in the Directory of Open Access Journals do not charge an APC at all (see footnote above on the terminology of ‘diamond OA’). Thus, alternative funding models for OA publishing which do not necessarily incentivize low acceptance thresholds exist [190].
